# On Tumor of Testis, Containing “Fœtal Remains,” with a Case

**Published:** 1866-03

**Authors:** W. H. VanBuren

**Affiliations:** Prof. of Anatomy, University of New York, &c., &c.


*On Tumor of Testis, containing "Fœtal Remains," with a case.* By W. H. VanBuren, M. D., Prof. of Anatomy, University of New York, &c., &c.

A male child, two years and five months old, was brought to me in the month of October, 1864, with a tumor of the scrotum, apparently involving the left testicle, about the size of a large hen's egg. The child was healthy at birth, and the swelling of the testis was not noticed until he was three months old, when a physician was consulted, who, regarding the disease as a hydrocele, passed a seton through the tumor in the form of a worsted thread. So much pain followed this operation that the seton was withdrawn in three hours, a glairy fluid escaping with some force, but in small quantity, and the tumor remaining hard, and subsequently growing harder under the very severe inflammation which followed. After the subsidence of the inflammation the tumor remained about at its original size, but somewhat harder, for nearly a year, when several abscesses formed and discharged themselves consecutively; and finally, after quite a large abscess had opened, a red, fungous mass protruded from its orifice, which gradually reached the size of an English walnut.

I found the tumor presenting fully one-half of its mass in the shape of this fungous protrusion, which was covered with unhealthy granulations discharging watery pus, hard to the touch, and occupying its lower and larger portions. Its upper portion, towards the spermatic cord, was generally smooth upon its surface, of a soft solid consistence, the skin adherent, and not very painful when grasped. It had never been painful except when the abscesses were forming, and when it was punctured. There were no enlarged glands in the groins. The child was weakly and pallid, and suffering from diarrhœa.

Dr. Valentine Mott had seen the case, suspected that the disease was malignant in its nature, and advised its removal.

I suggested to the father that he should take the child to the sea-side for a month, and pay close attention to his diet. At the end of this time his health was very much improved, but the tumor was unchanged. I was able to form no positive opinion as to its nature, but felt no doubt as to the propriety of its removal, and I therefore removed it by castration, in the usual manner.

The child made a rapid recovery after the operation, and I have heard, within a few days, that he is in excellent health and growing finely.

On examination of the tumor, after removal, the portion already described as a fungous protrusion, and which occupied the lower part of the tumor, was found partially covered at its lowermost surface by integument, and upon this integument, posteriorly, was a surface half an inch in diameter, thickly covered with hairs, some of them an inch in length, and presenting, under the microscope, the characteristic appearance of hairs from the head. Upon the surface of the protrusion was the orifice of a fistula, and on introducing a probe into the fistulous tract it came in contact with a very hard, smooth, apparently bony, surface. When laid open by the scalpel a cavity was disclosed about an inch in diameter, containing pus, and in contact with, and adherent to its walls, a fragment of bone, covered by periosteum resembling in shape a fragment of the body cf a fœtal lower jaw-bone. The length of this fragment of bone was five-eighths of an inch, its breadth three-eighths of an inch, and its thickness about the same. Implanted somewhat irregularly upon one of its margins were found four teeth, slightly altered from their normal shape, but distinctly recognizable as two incisors, one canine and one molar, in their normal relation, and appropriate in size to that of the fragment (of jaw-bone) in which they were implanted. On withdrawing the molar tooth from its alveolar socket, which was normal in its proportions, it was found to present the crown of a well formed molar, hollow within, and destitute of fangs. At the bottom of the alveolar socket the dental sac was distinctly visible, and protruding from its floor was the well formed surface of a second (permanent) molar tooth, which, when touched by a probe, was soft, and evidently not yet encrusted by enamel. It was with one of these teeth that the probe came in contact when introduced into the fistulous tract. Both the tooth and bone structure were examined microscopically, the former showing enamel prisms, and the latter the lacunæ and canaliculi of true bone.

Situated above the cavity, which inclosed the bone and teeth, was a second cavity containing turbid fluid, and lined by a smooth, apparently serous, membrane—the probable remains of the tunica vaginalis. In contact externally with the walls of this cyst was the testicle, normal in size and appearance, with the exception of an abscess, the size of a large pea, situated in its substance. The glandular elements of the testicle were recognized under the microscope. The mass of the tumor situated above and around the testicle, and constituting about one-half the tumor's bulk, was found under the microscope to consist of the elements of connective tissue, consolidated by inflammation.

The microscopic examination of the specimen was made by Dr. J. W. J. Gouley.

It is evident that the tumor is an example of that rare pathological condition known heretofore by the English as "fœtal remains in the testicle," and by the French as "*inclusion scrotale et testiculaire.*" The latter designation is the more intelligible of the two, as it indicates the nature of this curious growth, which is an imperfect effort at the production of a double monster, or "monstrosity by inclusion," or *fœtus in fœtu* of the older writers. It is described as one of the varieties under this latter head in Vrolik's classification of fœtal deformities, and he remarks that "it is most probable that the *fœtus in fœtu* is an incomplete effort to form a double monster." (Cyclopædia of Anatomy and Physiology, art. Teratology.) The term "monstrosity by inclusion" belongs to the great French teratologist, Geoffroy St. Hilaire, and has been adopted by Cruveilhier in his "Pathologie Generale," and applied to tumors containing fœtal remains which have occurred in different parts of the body, *e. g.*, in the perineum, over the sacrum, in the thoracic and abdominal cavities, the liver and ovaries, as well as in the scrotum.

This explanation of the pathological nature of these tumors has been more recently disputed by Lebert, who endeavors to include them in his class of "dermoid cysts," or misplaced growths of normal tissues. The pathological law under which all these growths are developed is thus stated by Lebert, (Traite d'Anat. Path., t. i., p. 260.) "That both simple and compound tissues, and even more complicated organs, are capable of developing themselves in parts of the body where normally they do not exist." This he considers that he has established, and its truth is generally admitted by pathologists. But Lebert does not entirely exclude the theory of inclusion, as will be inferred from the following quotation: "I have brought together three cases of dermoid cysts of the scrotum, and endeavored to establish the points of difference which distinguish them from true cases of fœtal inclusion occurring in this same locality—in which undoubted debris of the skeleton are recognizable." (V. ut supra, p. 257.)

The theory of inclusion of St. Hilaire has also been disputed by the latest authority on the subject, Dr. George Murray Humphry, lecturer on surgery and anatomy in the Cambridge University Medical School, the author of the article on "Diseases of the Male Organs" in Holmes' System of Surgery, who considers that Lebert's theory of "heterotopie plastique" is entirely sufficient to explain the nature of tumors connected with the testicle containing fœtal remains. (Holmes' Surgery, v. iv., p. 600.)

The question as to the real origin of these tumors appears, therefore, to be still an open one, and it may be stated succinctly as follows: Is a scrotal tumor, containing so-called "fœtal debris," the result of a local plastic effort determined by injury or inflammation, and liable to occur at any period of life; or is it the production of a fecundated Grœffian vesicle accidentally included in the scrotum of a twin fœtus, and thus arrested in its development, and of necessity congenital? This question is more curious than practically useful, for, as Dr. Humphry concludes, the only remedy for these tumors is to remove them by operation. Those desirous of pursuing it further will find it elaborately discussed by Lebert, (as above) by Cruveilhier in his Pathologie Generale, t. i. p. 370, and t. iii., p. 582, et seq., and by Verneuli in the paper referred to below.

It follows, if Cruveilhier is right, that tumors connected with the testicle of this character must be always congenital, and such appears to be the fact. M. Verneuil has collected all the authentic cases on record, to the number of ten in all, and treated the subject very ably and exhaustively in a series of papers published in the Archives Generale de Medicine in 1855. The earliest recorded of these cases is the only one of the ten in which the congenital character of the tumor is not clearly demonstrated. "A young man of quality, after exposure to sexual excitement, was seized with a sudden pain in the right testicle; this soon subsided, but shortly afterwards he discovered an unnatural growth connected with the testis, which rapidly increased to the size of the head of an infant of six months, and within the year was removed by a surgeon of Sisterton, France, named St. Donat. On opening the tumor, after its removal, it was found to contain the somewhat altered remains of a fœtal cranium; the testis was compressed and altered in appearance, and the fœtal remains seem to have been enclosed in a cyst attached externally to the testis." The case was transmitted by St. Donat to Pierre Amand, a member of the faculty of Paris, and published by Amand in a volume on Obstetrics, at Paris, in 1715.

In a case reported by Prochask "an otherwise well formed male infant was born with a small tumor in the groin, which was taken for a hernia. When three years old it commenced to grow, rapidly filled the scrotum, and in a few weeks reached as low as the middle of the thigh, when an abscess formed and discharged a fetid fluid, together with several portions of the skeleton of a fœtus, after which the child rapidly got well."

The following case, reported by Ollivier, (D'Angers) presents some features similar to mine: "Ovide-Emile Caze, well formed at his birth, was discovered by his parents, when a year old, to have the right testicle larger than the left, and six months later was operated upon by Dr. Capon, for hydrocele. A little serous fluid followed the puncture, but the testicle remained larger than before, so that two years afterwards another operation was talked of, but as the swelling was painless nothing was done. During his seventh year the testis, having reached three times its natural size, became painful, and an ulceration having taken place, a reddish mass protruded, in which Dr. Andre, having discovered a hard, white polished surface resembling a tooth, diagnosticated a tumor connected with the testis containing fœtal remains." The protrusion increasing it was tied off, and afterwards examined by Ollivier, and found to contain four teeth and a piece of spongy bone, contained, apparently, in a sort of cyst. The child, who was left mainly to nature, was thought likely to get entirely well. (Memoires sur la Monstruosite par Inclusion; Archives Generales de Medicine, t. xv., p. 540.)

In Velpeau's celebrated case, which occurred in La Charite Hospital, in Paris, whilst I was an externe in that institution, in 1840, the patient, who was 27 years of age, had a tumor the size of the fist on the right side of his scrotum, which had existed since his birth. It was painless, and presented several fistulous openings, from one of which a tuft of hair projected, and this circumstance suggested the true nature of the tumor. Velpeau made it a point of saving the testicle, which could be distinguished from the tumor, although closely connected with it, and this necessitated a long and difficult dissection. The tumor contained much fœtal debris and a number of easily recognizable bones of the fœtal skeleten. The patient died of purulent infection. The case is recorded in the Gazette Medicale de Paris, Feb. 15th, 1840.

In M. Verneuil's case, which occurred in the wards of M. Guersant, in the Children's Hospital of Paris, the fœtal debris were very carefully examined by the microscope, and, amongst other tissues, the histological elements of the gray substance of the brain were distinctly recognized.

Of the ten cases collected by M. Verneuil but two were diagnosticated; those of Andre and Velpeau. If the congenital character of these tumors is admitted, it constitutes their most valuable diagnostic feature. The diagnosis would lie between hernia, hydrocele, encephaloid cancer and tubercular disease of the testis. It would seem easy to exclude the two former, although two of the cases noted in this paper were mistaken for hydroceles.

Robert speaks of a case of congenital soft cancer of the testis; and I once saw a well marked case of syphilitic enlargement of the gland in a child of eighteen months, who also had periosteal swellings and other evidences of inherited disease.

It is not unlikely that there are other cases of this curious affection which have not yet been placed on record, and if this imperfect notice of the subject should lead to any further additions to our knowledge by eliciting unrecorded cases, or by rendering their nature more apparent, it will have attained its object.—*New York Medical Journal.*

